# The antioxidative defense system is involved in the premature senescence in transgenic tobacco (*Nicotiana tabacum NC89*)

**DOI:** 10.1186/s40659-016-0088-1

**Published:** 2016-07-02

**Authors:** Yu Liu, Lu Wang, Heng Liu, Rongrong Zhao, Bin Liu, Quanjuan Fu, Yuanhu Zhang

**Affiliations:** State Key Laboratory of Crop Biology, College of Life Sciences, Shandong Agricultural University, 61 Dai Zong Street, Tai’an, 271018 Shandong People’s Republic of China; Shandong Institute of Pomology, 66 Long Tan Road, Tai’an, 271018 Shandong People’s Republic of China

**Keywords:** Tobacco, Senescence, Transcriptome, Reactive oxygen species (ROS)

## Abstract

**Background:**

α-Farnesene is a volatile sesquiterpene synthesized by the plant mevalonate (MVA) pathway through the action of α-farnesene synthase. The *α*-*farnesene synthase 1* (*MdAFS1*) gene was isolated from apple peel (var. *white winter pearmain*), and transformed into tobacco (*Nicotiana tabacum* NC89). The transgenic plants had faster stem elongation during vegetative growth and earlier flowering than wild type (WT). Our studies focused on the transgenic tobacco phenotype.

**Results:**

The levels of chlorophyll and soluble protein decreased and a lower seed biomass and reduced net photosynthetic rate (Pn) in transgenic plants. Reactive oxygen species (ROS) such as hydrogen peroxide (H_2_O_2_) and superoxide radicals (O_2_^·−^) had higher levels in transgenics compared to controls. Transgenic plants also had enhanced sensitivity to oxidative stress. The transcriptome of 8-week-old plants was studied to detect molecular changes. Differentially expressed unigene analysis showed that ubiquitin-mediated proteolysis, cell growth, and death unigenes were upregulated. Unigenes related to photosynthesis, antioxidant activity, and nitrogen metabolism were downregulated. Combined with the expression analysis of senescence marker genes, these results indicate that senescence started in the leaves of the transgenic plants at the vegetative growth stage.

**Conclusions:**

The antioxidative defense system was compromised and the accumulation of reactive oxygen species (ROS) played an important role in the premature aging of transgenic plants.

**Electronic supplementary material:**

The online version of this article (doi:10.1186/s40659-016-0088-1) contains supplementary material, which is available to authorized users.

## Background

α-Farnesene is a volatile plant sesquiterpene. It also acts as a semiochemical that can affect the behavior of some insect species [1, 2]. α-Farnesene can accumulate in apple peel, and the oxidative production of α-farnesene is associated with the development of scald symptoms [3, 4], a physiological disorder of apple and pear fruits [5, 6]. α-Farnesene synthesis mainly occurs via the cytosolic mevalonic acid (MVA) pathway, which is initiated by the first rate-limiting enzyme, 3-hydroxy-3-methylglutaryl-CoA reductase (HMGR) [7]. The other important step in the α-farnesene synthesis pathway involves farnesyl diphosphate synthase (FPS). FPS catalyzes the condensation of geranyl diphosphate (GPP) and isopentenyl diphosphate (IPP) to produce farnesyl diphosphate (FPP), which is the immediate precursor of sesquiterpenes [8]. In the final step of synthesis, α-farnesene synthase uses FPP as the substrate to catalyze the synthesis of α-farnesene. Pechous and Whitaker (2004) cloned the *α*-*farnesene synthase 1* (*AFS1*) gene of the ‘Law Rome’ apple and expressed it in bacteria [9]. When farnesyl diphosphate (FPP) was supplied as the substrate for the bacterially expressed recombinant enzyme, α-farnesene was synthesized.

Terpenoids play crucial roles in plant defense, growth, and development. Genetic engineering of key genes involved in the terpene synthesis pathway has generated mutants and transgenic plants with altered growth and development. For example, the *hmg1* mutant exhibits dwarfing, early senescence, sterility, and earlier induction of the *senescence*-*associated 12* (*SAG12*) gene compared to wild-type (WT) plants [10]. In *Arabidopsis thaliana*, farnesyl diphosphate synthase 1 (FPS1) and farnesyl diphosphate synthase 2 (FPS2), which encode cytosolic FDP synthase, are differentially expressed [11]. Compared to WT, overexpression of *A.thalianaFPS1* results in a cell death/senescence-like phenotype, which grew less vigorously than wild-type plants, and premature induction of the *SAG12* gene [12]. Overexpression of *A. thaliana FPS2* leads to the synthesis of several novel sesquiterpenes, including E-β-farnesene, and transgenic plants show enhanced growth [13]. Of the three functional FPPS in maize, *FPPS3* is induced by herbivory, and is essential to the production of the volatile sesquiterpenes, including β-caryophyllene [14]. Sesquiterpenes play an important role in plant physiological and ecological functions such as scavenging for reactive oxygen species (ROS), stabilizing membrane structure, and inhibiting bacterial growth [15, 16]. Treatment of *Aquilaria sinensis* (Lour.) cell culture suspensions with hydrogen peroxide (H_2_O_2_) induces the production of sesquiterpenes, stimulates programmed cell death (PCD), and increases salicylic acid (SA) accumulation. These results indicate potential interactions between sesquiterpene synthesis and programmed cell death (PCD) during *A. sinensis* (agarwood) formation [17]. In plants, terpenes are synthesized via the mevalonate (MVA) pathway in the cytosol and peroxisomes, and the 2-C-methyl-d-erythritol 4-P/1-deoxy-d-xylulose 5-P (MEP) pathway in plastids [18]. The two pathways are capable of exchanging intermediates [19]. In the MEP pathway, disruption of the *1*-*deoxy*-*d*-*xylulose*-*5*-*phosphate reductoisomerase* (*DXR*) gene leads to biosynthetic deficiency of photosynthetic pigments, GAs and ABA, resulting in developmental abnormalities [20]. Monoterpenes are volatile terpenes synthesized by the MEP pathway. Monoterpenes also play key roles in plant defense and apoptosis-like cell death [21–23].

Leaf senescence is characterized by degradation of the chlorophyll, photosynthetic proteins and other macromolecules, conversion of peroxisomes into glyoxysomes, and increased production of ROS [24]. ROS are a central element of the senescence process, which is toxic to plant cells [25]. Excessive production of ROS causes necrosis via programmed cell death [26]. The chloroplast is the most important organelle in ^1^O_2_, production and is also regarded as the major intracellular producer of partially reduced oxygen species such as H_2_O_2_ and O_2_^·−^ [27]. Chloroplasts, which are a rich source of nitrogen (N), are the first organelles that are dismantled during senescence [28]. Nutrient signaling also plays important roles in leaf senescence. For example, leaf senescence is induced when sugar levels exceed or decrease beyond acceptable threshold levels [29, 30]. Sugar-induced senescence is particularly important in conditions of low nitrogen availability and may also play a role in light signaling [31, 32].

Overexpression of *α*-*farnesene synthase 1* (*MdAFS1*) in transgenic tobacco plants has been associated with an unexpected phenotype, specifically accelerated stem elongation and early flowering, compared to WT plants. Therefore, in this study, transcriptome analysis of transgenic tobacco plants was conducted to study the differentially expressed genes. We concluded that the antioxidative defense system is compromised, and accumulation of ROS activates the senescence process. In the future, we anticipate that the transcriptome database will be a valuable resource for improved understanding of the molecular basis for alterations in plant growth and development.

## Results

### Identification of transgenic plants

**Fig. 1:**
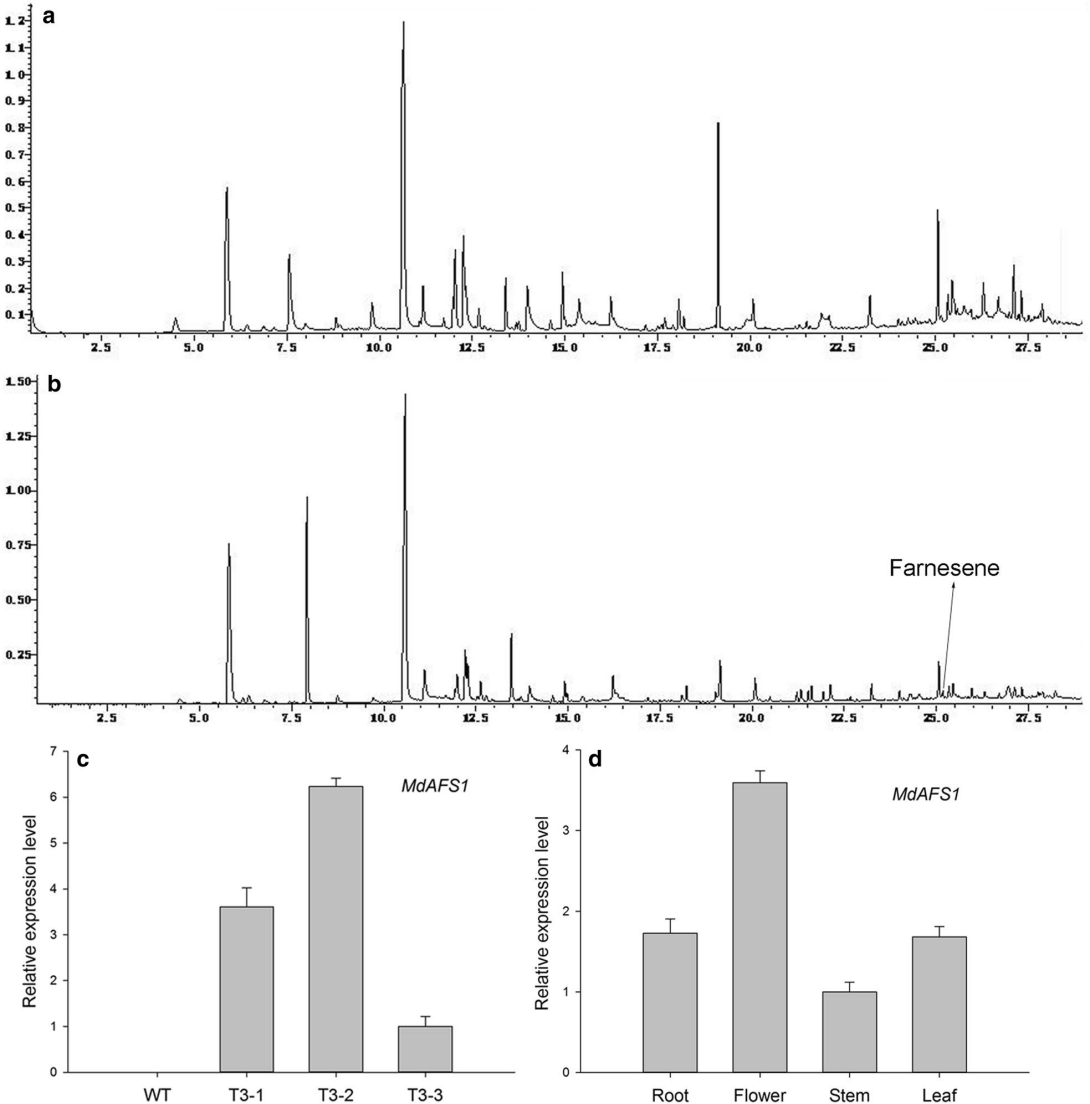
Identification of transgenic plants by qRT-PCR and GC–MS. **a** GC–MS analysis of terpenes of WT plants. **b** GC–MS analysis of terpenes of transgenic plants. **c** *MdAFS1* transcript in different transgenic plants. **d** *MdAFS1* transcript in different parts of transgenic plants. The *MdAFS1* transcript level was normalized to 18S RNA expression. The standard error of the mean of three biological replicates (nested within three technical replicates)

Three third generation lines were selected for identification of transgenic plants at the transcript level. The relative *MdAFS1* mRNA levels of the transgenic lines were average, higher, and lower in T3-1, T3-2 and T3-3, respectively (Fig. 1c). Expression levels of *MdAFS1* varied in different parts of the transgenic plants; it had a higher transcript level in the flowers (Fig. 1d). So, flowers were selected as experimental materials for GC–MS analysis of the terpenes. The release of farnesene (0.41 %) was detected in the flowers of transgenic plants (Fig. 1a, b). The chemical formula of farnesene is C_15_H_24_ and the molecular weight is 204. In addition to this, β-myrcene (0.34 %), linalool (5.92 %), and caryophyllene (2.98 %) were detected in WT plants. The release of linalool (2.3 %) and caryophyllene (1.71 %) was detected in the flowers of transgenic plants (Table 1).

**Table 1:** GC-MS analysis of tobacco terpenes

Retention time (min)	Compound formula	Compound name	Relative percentage content (%)	
			WT	T3-2
16.317	C_10_H_16_	β-Myrcene	0.34	–
19.127	C_10_H_16_	Linalool	5.92	2.3
25.064	C_15_H_24_	Caryophyllene	2.98	1.71
25.175	C_15_H_24_	Farnesene	–	0.41

“–”  Indicates that this compound was not tested

### Plant phenotype analysis

**Fig. 2:**
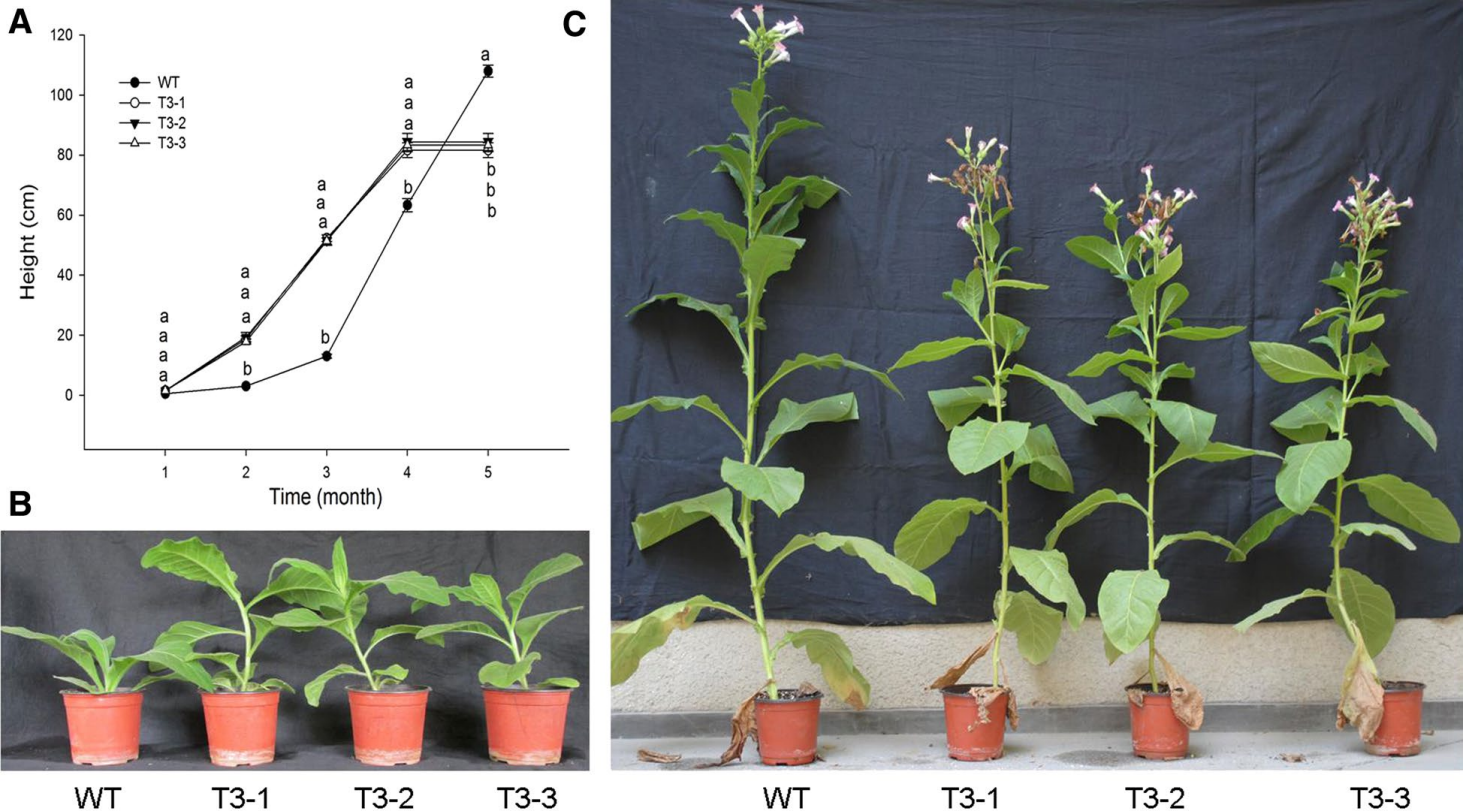
Phenotypic variation of wild-type (WT) and transgenic plants at different developmental stages. **A** Variation in the height of WT and transgenic plants at different developmental stages, each *line* is the mean of five replicates.* Different* *letters* indicate statistically significant differences at *P* ≤ 0.05, **B** eight-leaf period, and **C** filling period

To determine growth differences in the WT, T3-1, T3-2 and T3-3, plant height, leaf area, and seed biomass were measured. At 8 weeks of age, the stem of transgenic plants had accelerated elongation with lengths reaching about 21 cm, whereas WT stems were approximately 2.0 cm in length (Fig. 2B). The transgenic plants flowered at about 10 weeks of age and had mature seeds at 18 weeks. At 18 weeks, the WT plants were still in full bloom. The heights of the tobacco plants were as follows: 108.0 cm, 81.7 cm, 84.0 cm and 83.3 cm for WT, T3-1, T3-2 and T3-3, respectively (Fig. 2C). Based on changes in height throughout the developmental process, the transgenic plants had faster development (Fig. 2A). At 8 weeks old, the transgenic plants showed no change in leaf biomass, a fivefold increase in stem biomass, and a twofold increase in root biomass (Table 2). There were 9–10 leaves per transgenic plant, whereas WT plants had 8 leaves. Leaf area was measured at different locations on transgenic and WT plants. Transgenic plants showed no changes in total leaf area but the functional leaf area significantly decreased and the upper leaf area significantly increased (Table 3).

**Table 2:** Biomass of tissues from wild-type (WT) and transgenic (T) plants

Biomass (g)	WT	T3-1	T3-2	T3-3
Root	1.73 ± 0.09b	3.26 ± 0.62a	3.06 ± 0.10a	3.65 ± 0.76a
Stem	2.19 ± 0.09b	10.84 ± 1.90a	10.76 ± 0.63a	11.46 ± 1.90a
Leaf	21.33 ± 4.33a	20.93 ± 2.82a	20.63 ± 1.52a	22.57 ± 2.61a
Total	25.25 ± 2.70b	35.03 ± 12.90a	34.46 ± 4.60a	37.67 ± 17.53a

Each line represents the mean of five replicates.* Different letters* indicate statistically significant differences at *P* ≤ 0.05 in same parts of wild-type (WT) and transgenic (T) plants

**Table 3:** Leaf area of WT and transgenic (T) plants

Lines	1 (cm^2^)	2 (cm^2^)	3 (cm^2^)	4 (cm^2^)	5 (cm^2^)	6 (cm^2^)	7 (cm^2^)	8 (cm^2^)	9 (cm^2^)
WT	28.8 ± 5.0a	62.8 ± 13.3a	82.9 ± 14.7a	158.5 ± 14.6a	154.8 ± 12.4a	112.8 ± 5.4a	77.9 ± 8.9a	25.3 ± 7.6b	
T3-1	26 ± 8.4a	59.2 ± 21.0a	97.3 ± 4.8a	96.8 ± 1.1b	132.9 ± 2.6b	103.0 ± 6.5a	89.5 ± 7.0a	56.4 ± 14.2a	18.7 ± 6.5
T3-2	30.2 ± 1.0a	67.5 ± 2.7a	89.9 ± 10.2a	124.6 ± 5.6b	133.5 ± 2.7b	111.4 ± 12.0a	86.1 ± 11.6a	53.2 ± 0.3ab	23.2 ± 2.9
T3-3	24.8 ± 3.2a	58.0 ± 8.4a	81.4 ± 1.9a	116.7 ± 11.9b	128.9 ± 6.7b	116.1 ± 1.5a	88.8 ± 6.2a	53.5 ± 10.5ab	31.1 ± 16.3

Each line represents a mean of five replicates.* Different letters* indicate statistically significant differences at *P* ≤ 0.05 in same leaf position of wild-type (WT) and transgenic (T) plants

### The transgenic plants have reduced seed biomass

Seed biomass was considered an important indicator of reproductive growth. One capsule of tobacco (*NC89*) contains 1500–2000 grains. Though the weight per 1000 grains increased by 117.1, 115.6 and 111.4 %, the number of capsules decreased by 66.7, 70.8 and 76.9 % in the T3-1, T3-2, and T3-3 lines, respectively, compared to WT plants (Table 4). Given these results, the transgenic plants produced less seed biomass than WT plants.

**Table 4:** Seed biomass of WT and transgenic (T) tobacco plants

Biomass	WT	T3-1	T3-2	T3-3
Capsule (number)	24.0 ± 1.0a	16.0 ± 1.0b	17.0 ± 3.0b	16.3 ± 2.3b
Weight per 1000 grains (mg)	76.2 ± 1.5c	89.2 ± 3.5a	88.1 ± 1.8a	84.9 ± 2.2b

Tobacco seeds dried to constant weight were used in this experiment. Values shown are means ±  SE ( five biological replicates is presented)

Different letters indicate statistically significant differences at P ≤ 0.05

### Physiological parameters related to senescence

**Fig. 3:**
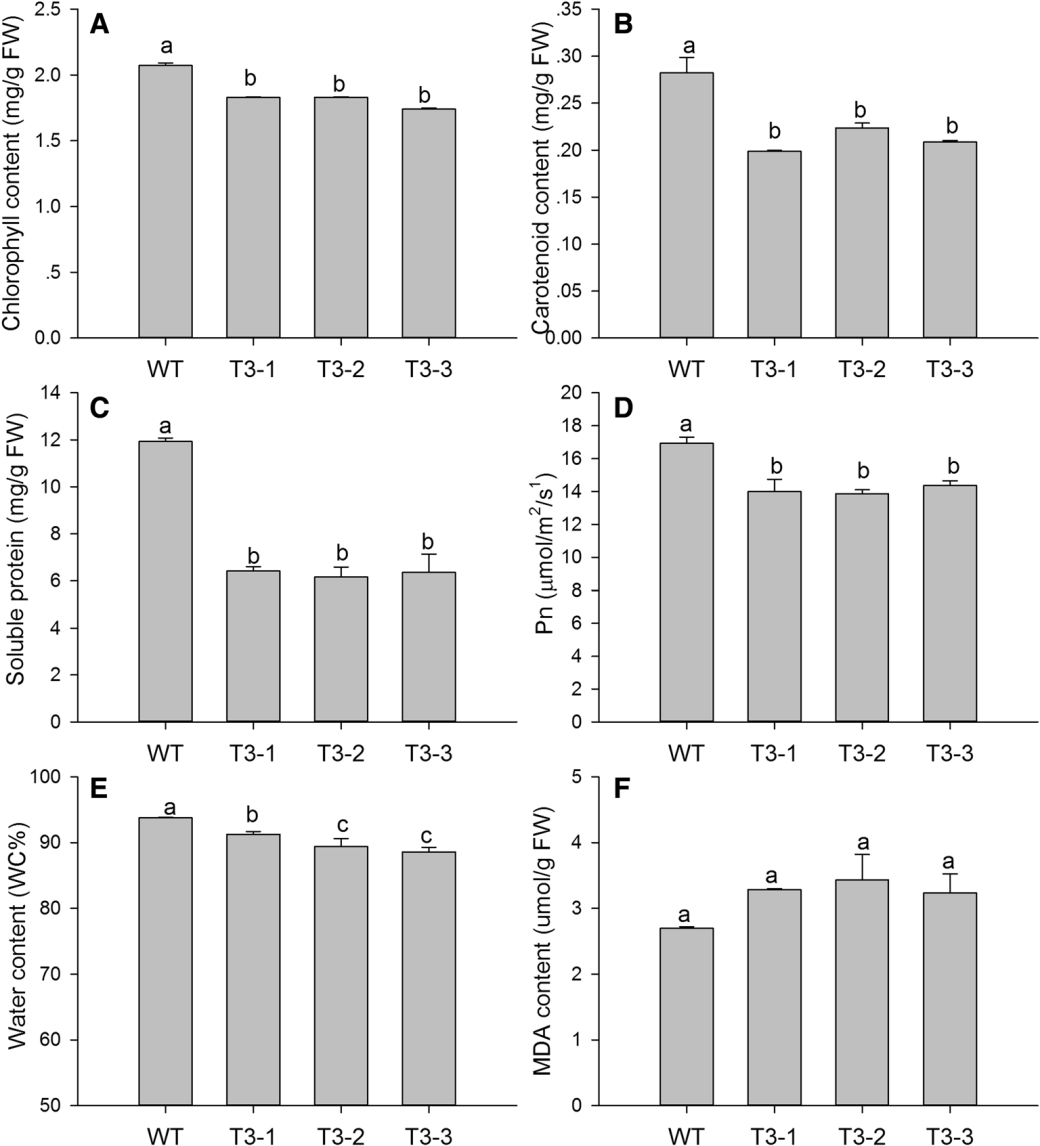
Measurement of senescence-related physiological parameters in WT and transgenic plants. **A** Chlorophyll content, **B** carotenoid content, **C** soluble protein content, **D** net photosynthetic rate, **E** shows water content, and **F** signifies malondialdehyde (MDA) content. Data are mean ± SE (n = 5, five biological replicates per *line*).* Different* *letters* indicate statistically significant differences at *P* ≤ 0.05

Leaf senescence generally accelerates chlorophyll degradation and cell death [33, 34], which occur simultaneously with protein degradation. For these reasons, chlorophyll and soluble protein levels were measured. The T3-1, T3-2 and T3-3 plants showed a decrease in the content of chlorophyll *a* by 87.1, 83.9 and 78.2 %; chlorophyll *b* decreased by 93.7, 86.3 and 80.4 %; the total chlorophyll (a + b) content decreased by 88.2, 88.3 and 84 % (Fig. 3A) and carotenoid content decreased by 70.5, 79.2 and 73.9 %, respectively (Fig. 3B). Soluble protein levels decreased significantly, by 53.8, 51.6 and 53.3 % in T3-1, T3-2 and T3-3, respectively (Fig. 3C). Chlorophyll and photosynthesis proteins are important elements in photosynthesis. The value of Pn decreased significantly by 82.7, 81.9 and 84.8 % in three transgenic lines (Fig. 3D). Water content showed a significant reduction (Fig. 3E). Malondialdehyde (MDA) is the decomposition product of membrane lipid peroxidation, and it accumulates in senescent leaves. MDA content increased by 121.7, 127.1 and 119.7 % in the T3-1, T3-2, and T3-3 lines, respectively (Fig. 3F).

### Differentially expressed unigenes in leaf transcriptome data are related to senescence

**Fig. 4:**
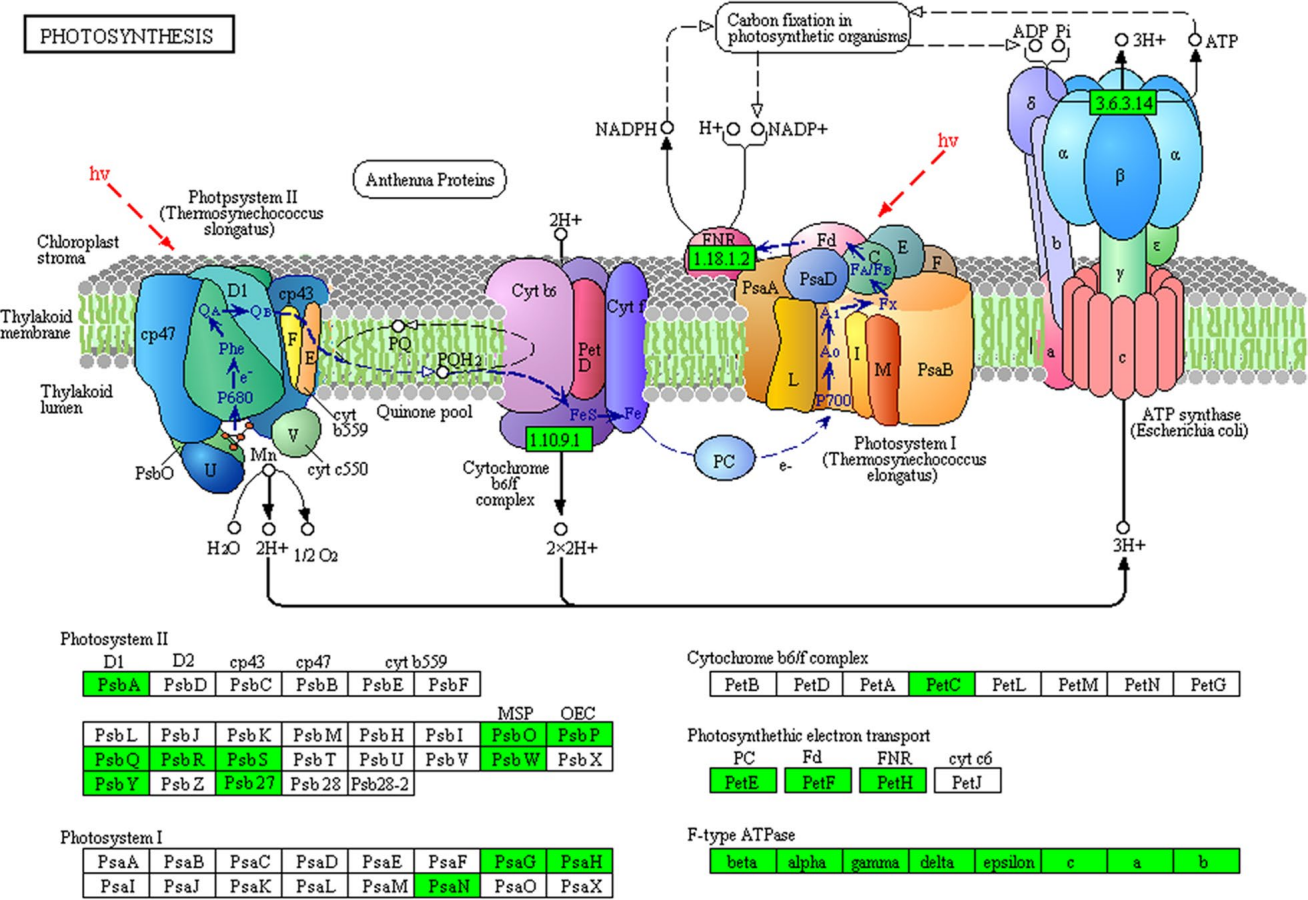
Kyoto encyclopedia of genes and genomes (KEGG) pathway analysis of photosynthesis. *Green* represents downregulated unigenes (*P* ≤ 0.01, ratio ≤0.5). *psbS* chloroplast photosystem II 22 kDa component, *psaN* photosystem I reaction center subunit, *psbY* photosystem II core complex proteins, *psbP* photosystem II oxygen-evolving enhancer protein 2, *petC* Rieske FeS precursor protein 2, *atpG* gamma subunit of ATP synthase, *petE* plastocyanin A, *psb27* photosystem II Psb27 protein, *psbO* chloroplast PsbO4 precursor, *psaH* photosystem I reaction centre subunit, *psaG* photosystem I reaction center V, *petF* ferredoxin, *atpF* ATP synthase subunit b, *psbW* photosystem II PsbW protein, *petH* ferredoxin-NADP reductase, *psbQ* photosystem II oxygen-evolving enhancer protein 3, *psbR* photosystem II 10 kDa polypeptide, *psbA* photosystem II protein D1

The molecular mechanism underlying leaf senescence in transgenic tobacco was studied using transcriptome analysis of 8-week-old plants. De novo transcriptome assembly generated a total of 249,185 unigenes. The total number of differentially expressed transcripts (*P* ≤ 0.01, ratio ≥2 or ≤0.5) was 5835, of which 2028 were upregulated and 3807 were downregulated in transgenic plants. The expression levels of six differentially expressed unigenes and three antioxidant enzyme genes were analyzed using qRT-PCR to test the reliability of the transcriptome database. The results were consistent with the transcriptome data (Additional file 1: Fig. S1). To classify the predicted functions of the unigenes, nucleotide and protein databases were used. GO and KEGG analyses showed that some differentially expressed unigenes were related to leaf senescence. For example, ubiquitin-mediated proteolysis, cell growth, and death were upregulated unigenes. However, antioxidant enzymes, nitrogen metabolism, photosynthesis, and carotenoid biosynthesis were downregulated unigenes (Additional files 2, 3). A total of 87 downregulated unigenes of the photosynthesis signaling pathway are presented in Fig. 4. All transcripts of tobacco (*N. tabacum* NC89) have been deposited to GenBank as Accession Number GDGU00000000.

### Accumulation of reactive oxygen species (ROS) in transgenic plants

**Fig. 5:**
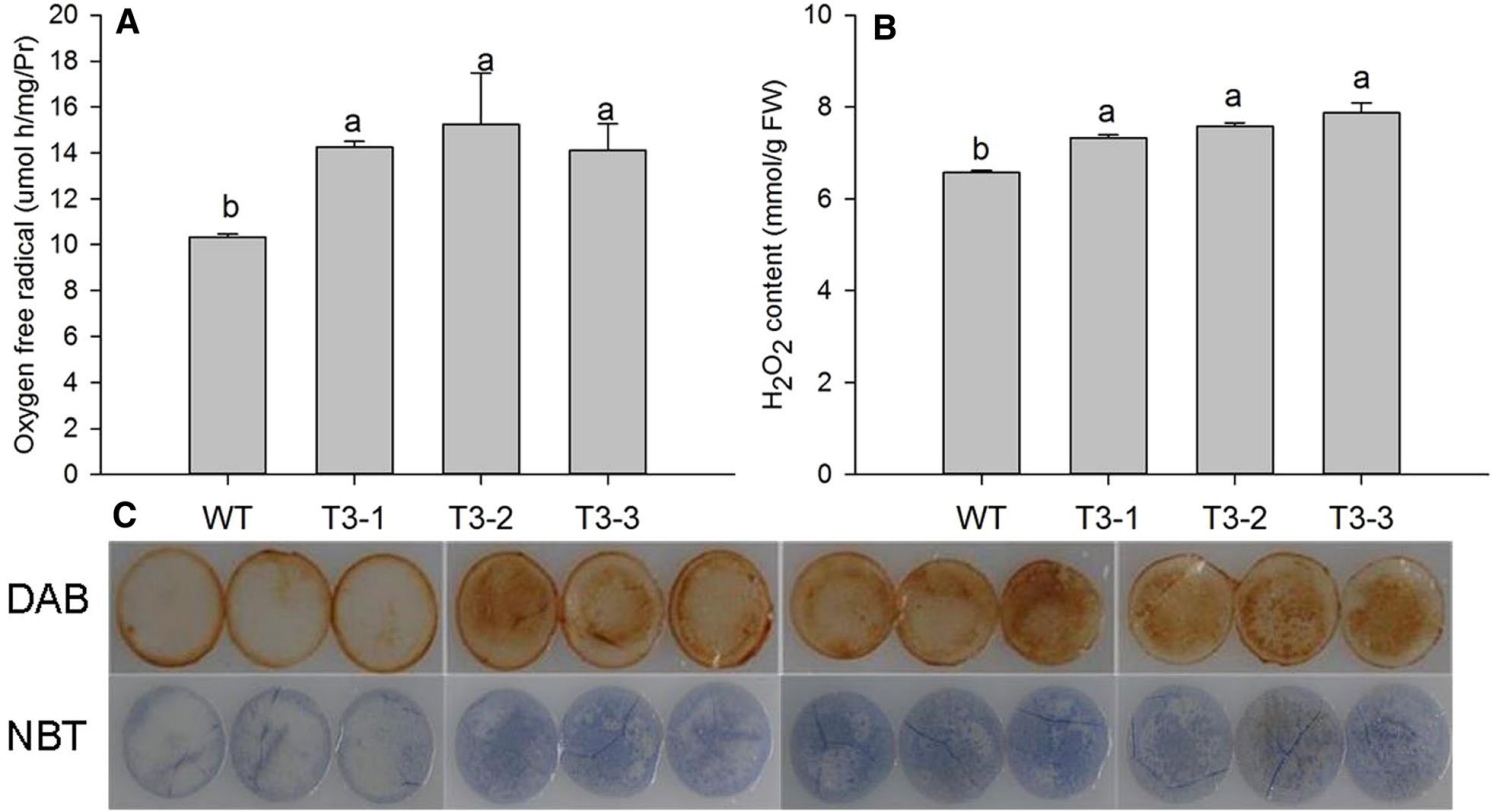
H_2_O_2_ levels and production rate of O_2_^·−^ in WT and transgenic plants. Data are expressed as the mean ± SE (n = 5, five biological replicates per *lines*).* Different* *letters* indicate statistically significant differences at *P* ≤ 0.05. **a** represents the content of H_2_O_2_. **b** indicates production rate of oxygen free radical. **c** shows histochemical staining of H_2_O_2_ and O_2_^·−^

As indicators of oxidative stress and leaf senescence, the levels of O_2_^·−^ and H_2_O_2_ in leaf tissues were measured under controlled conditions. Transgenic plants, T3-1, T3-2 and T3-3, had increases in the production rate of O_2_^·−^ by 138.0, 147.5 and 136.6 % (Fig. 5A), and H_2_O_2_ levels increased by 111.5, 115.3 and 119.8 %, respectively (Fig. 5B). Histochemical staining can detect the accumulation of reactive oxygen species (ROS); deeper 3,3′-diaminobenzidine (DAB) and nitroblue tetrazolium (NBT) staining was seen in transgenic plants. These findings were consistent with the ROS content results.

### Antioxidant enzyme activity and gene expression analysis

**Fig. 6:**
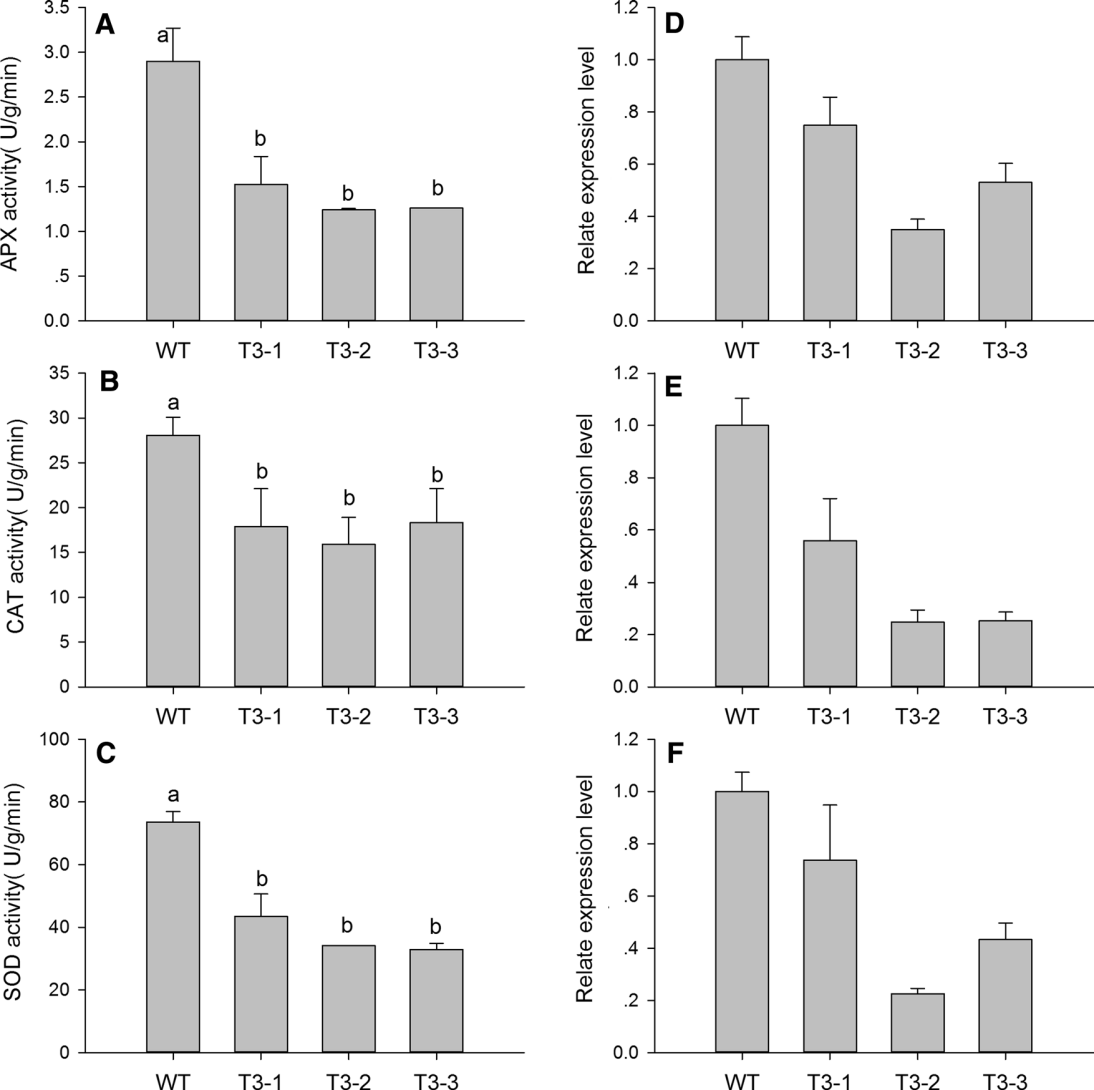
Activity and expression analysis of antioxidant enzymes in the WT, T3-1, T3-2, and T3-3 tobacco plants. **A** and **D** represent ascorbate peroxidase (APX), **B** and **E** represent catalase (CAT), and **C** and **F** represent superoxide dismutase (SOD). 18S RNA (GenBank Accession Number: AJ236016) was used as a housekeeping gene. The gene names and primers used for qRT-PCR analysis are presented in Additional file 6. Five biological replicates were used for each *line* to study of antioxidant enzyme activity. The standard error of the mean of three biological replicates (nested within three technical replicates) is represented by the *error bar*s in qRT-PCR analysis

Antioxidant enzymes participated in the scavenging of ROS. However, the activity of three antioxidant enzymes in T3-1, T3-2 and T3-3 decreased significantly, APX by 52.6, 42.9 and 43.4 %; CAT by 63.7, 56.6 and 65.3 %; and SOD by 59.1, 46.4 and 44.7 %, respectively (Fig. 6). The expression of antioxidant enzyme genes were consistent with the enzyme activity, which were downregulated in transgenic plants.

### Enhanced sensitivity of transgenic plants to DCMU treatment

**Fig. 7:**
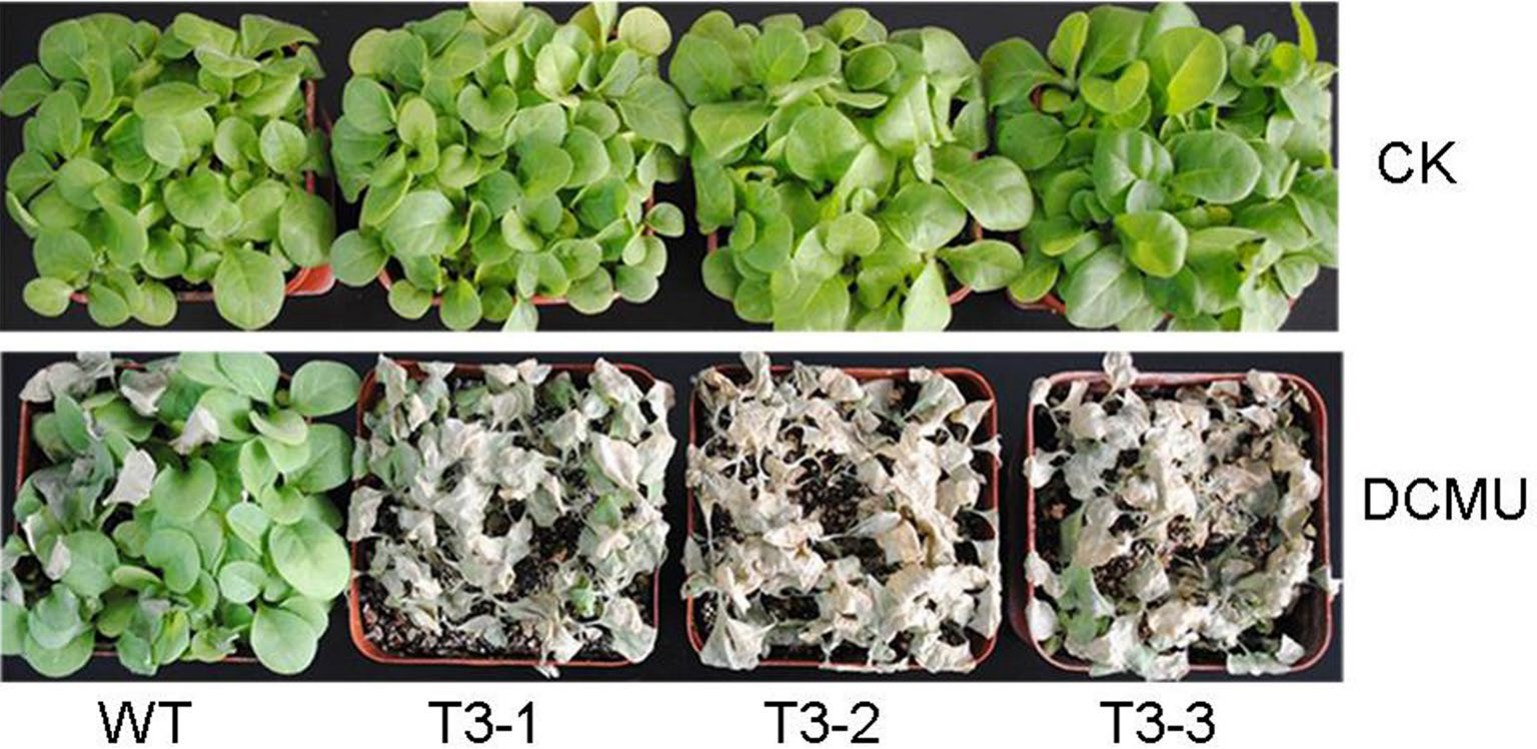
Enhanced sensitivity of transgenic plants to oxidative stress. The 3-week-old plants were treated with 70 μM DCMU for 10 days

The 3-(3,4-dichlorfenyl)-1,1-dimethylkarbonyldiamid (DCMU) molecule is an electron transfer inhibitor that acts during photosynthesis. Exogenous DCMU treatment induces production of ROS in the chloroplasts. Three week old plants were treated with 70 μM DCMU. After 10 days, all the transgenic plants had died, but the WT plants were still alive (Fig. 7). This result indicated that the transgenic plants were more sensitive to oxidative stress.

### Expression of senescence marker genes

**Fig. 8:**
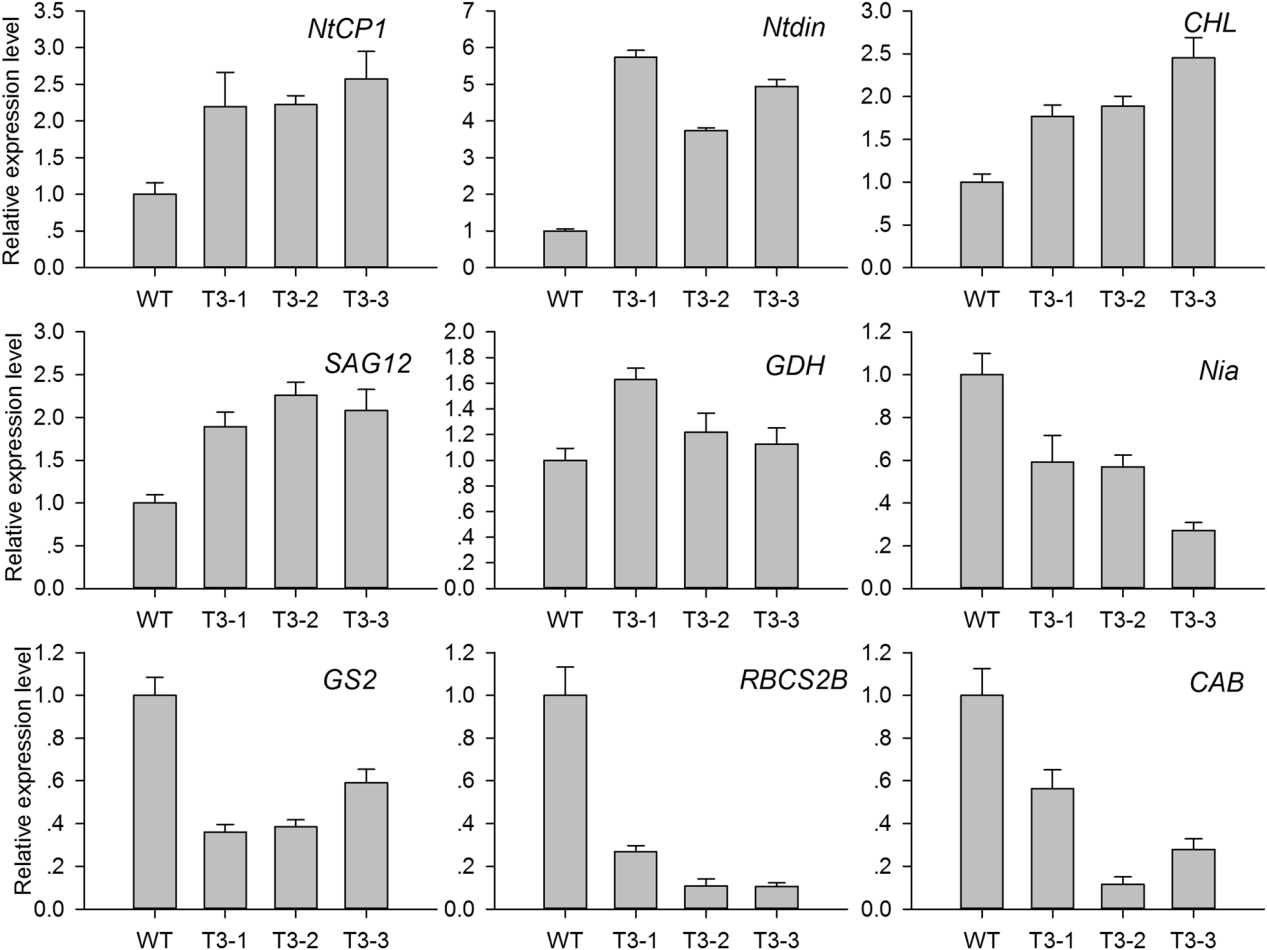
Expression analysis of senescence marker genes in WT and transgenic plants. *NtCP1* cysteine protease, *SAG12* cysteine protease, *Ntdin* a tobacco senescence-associated gene, *CHL* chlorophyllase, *GDH* glutamate dehydrogenase, *Nia* nitrate reductase, *GS2* chloroplastic glutamine synthetase, *RBCS2B* RuBisCO small subunit, *CAB* chlorophyll *a/b* binding protein. 18S RNA (GenBank Accession Number: AJ236016) was used as housekeeping gene. The gene names and primers used for qRT-PCR analysis are shown in Additional file 6. The standard error of the mean of three biological replicates (nested within three technical replicates) is represented by the *error bars* in qRT-PCR analysis

Based on the leaf senescence database (LSD, http://www.eplantsenescence.org/) and the typical characteristics of leaf senescence, senescence-related downregulated genes were selected, including chlorophyll *a/b* binding protein (*CAB*), ribulose-1,5-bisphosphate carboxylase-oxygenase small subunit (*RBCS2B*), nitrate reductase (*Nia*), and chloroplastic glutamine synthetase (*GS2*). The upregulated genes included cysteine proteinase (*NtCP1* and *SAG12*), glutamate dehydrogenase (*GDH*), chlorophyllase (*CHL*), and *Ntdin*. qRT-PCR confirmed that *CAB*, *RBCS2B*, *Nia*, and *GS2* were downregulated and that *NtCP1*, *SAG12*, *GDH*, *CHL*, and *Ntdin* were upregulated (Fig. 8). The transcript levels of these senescence marker genes are consistent with the premature senescence characteristic.

## Discussion

Transgenic plants showed a distinct premature senescence phenotype. The stems of transgenic plants rapidly elongated compared to those of WT plants. This occurred during the 7 week interval between first true leaf appearance to emergence of flower primordia (Fig. 2A). Transgenics had early flowering at about 50 days which was associated with a shorter vegetative period. Tobacco plants exhibited successive leaf senescence, starting from the basal leaves and advancing toward the apical leaves. At the filling period, which generally occurs in 20 week-old plants, the T3-1, T3-2 and T3-3 plants had at least two dead basal leaves, whereas WT plants only had one dead basal leaf (Fig. 2C). Changes in transgenic plant phenotype were indicative of premature senescence. Meanwhile, the transgenic plants developed new stems at the upper part of the plant. This phenomenon might be related to weak sink strength, which causes a slower progression of senescence in tobacco [35]. The new collateral delayed the death of the whole plant. Tobacco senescence is largely independent of floral development making it a model plant species for research [36]. However, leaf senescence in transgenic plants began during vegetative growth.

The main physiological purpose of leaf senescence is nutrient salvage involving the hydrolysis of macromolecules and their subsequent remobilization to other plant parts [37, 38]. We speculated that transgenic tobacco leaves activated the nutrient salvage program. Carbohydrates were temporarily stored in the stem and root tissues during vegetative growth, resulting in an increase the biomass of stems and roots (Table 2). This allocation of nutrients is beneficial to carbohydrate reserves and to the reduction of energy consumption [39], thereby remobilizing reproductive growth [28, 40]. At the reproductive stage, the premature senescent leaves of transgenic plants appeared to remobilize nutrients to the limited number of seeds, and increased the weight per 1000 grains. However, the decrease in total seed biomass is the result of senescence of transgenic plants.

Leaf senescence can be triggered by high C:N ratios [41, 42]. Glucose and sucrose repress the transcription of photosynthetic genes, probably via *hexokinase*, which acts as a sugar sensor, and by sugar phosphorylation, which mediates carbohydrate signal transduction [43]. Overexpression of *Arabidopsishexokinase* in tomato plants induces rapid senescence [44]. In contrast, antisense or *hexokinase* mutants exhibit a delayed senescence phenotype, suggesting that *hexokinase* is involved in senescence regulation [45]. In the present study, *hexokinase* is an upregulated unigene, which is likely to be involved with inducing senescence. In nitrogen metabolism, 62 transcripts were downregulated (Additional file 2). The expression of genes involved in primary nitrogen assimilation such as *GS2* (chloroplastic glutamine synthetase) and *Nia* (nitrate reductase) were repressed; in contrast, *GDH* (glutamate dehydrogenase) mRNA accumulation was increased (Fig. 8). This suggests that primary nitrogen assimilation was suppressed, and nitrogen remobilization was activated in transgenic plants. After treatment of tobacco leaf discs with hydrogen peroxide (H_2_O_2_) GDH mRNA accumulated, and GS2 mRNA decreased [46]. So the expression of GDH and GS2 also suggests the accumulation of ROS in transgenic plants. In addition, nitrogen is major element of soluble protein. Soluble protein degradation was indicative of lower nitrogen content (Fig. 3C).

Leaf senescence occurs during oxidative stress, which can be induced by overproduction of ROS [47]. Plants cope with oxidative stress using antioxidative systems including antioxidative enzymes such as glutathione peroxidases, dehydroascorbate reductase, catalase (CAT), superoxide dismutase (SOD), and ascorbate peroxidase (APX), and antioxidative metabolites such as ascorbate, glutathione, tocopherol, and carotenoids [48, 49]. In our study, the lower carotenoids, polyphenols, and flavonoid level could weaken their recognized roles in protecting photosystems from oxidative stress (Fig. 3B; Additional file 4: Fig. S2). Because SOD is responsible for dismutation of O_2_^·−^, it generates H_2_O_2_, which is subsequently scavenged by CAT and APX. The lower activity of antioxidative enzymes reduces effective ROS scavenging and ROS accumulate in transgenic plants (Figs. 5, 6). DCMU treatment also indicated that the transgenic plants suffered from severe oxidative stress. ROS such as O_2_^·−^ and H_2_O_2_ have also been implicated as age-associated factors that trigger leaf senescence which in turn enhances membrane lipid peroxidation [50]. There was detectable, but not significant, accumulation of MDA in transgenic plants (Fig. 3F). It is possible that the transgenic plants were still undergoing vegetative growth, which prevented the membrane lipid peroxidation.

Reactive oxygen species (ROS) induced plastid damage; chloroplast ROS influenced leaf senescence, and determined the viability and longevity of green tissues [51]. Downregulated genes are involved in senescence; *CAB* and *RuBisCO* small subunit, are responsible for the regulation of photosynthetic proteins. Chlorophyllase (CHL) operates the chlorophyll degradation pathway. The accumulation of *CHL m*RNA controls the release of ROS from the thylakoid membrane, which then initiates senescence. In addition to the above, relatively lower leaf water content led to a reduction in water potential, stomatal closure, and lower CO_2_ levels in aging mesophyll tissues, thereby reducing the net photosynthetic rate (Fig. 3E; Additional file 5: Fig. S3). The degradation of chlorophyll and photosynthetic proteins in transgenic plants suppressed the net photosynthetic rate (Fig. 3). A decline in photosynthetic capacity is a major feature of senescent leaves. In photosynthetic reactions, photosystem II, photosystem I, cytochrome b6/f complex, photosynthetic electron transport, and F-type ATPase distributed 87 downregulated unigenes in transgenic plants (Fig. 4). This further revealed that photosynthesis was suppressed in transgenic plants.

Proteins in plant leaves are constantly engaged in stable turnover. With plant leaf senescence, this dynamic balance is disturbed and leads to protein degradation. Protein degradation generally occurs via the ubiquitin–proteasome system. GO analysis indicated that several ubiquitin-mediated proteolysis unigenes were upregulated in transgenic plants (Additional file 2). In plants, a portion of senescence-associated proteases are localized in senescence-associated vacuoles to degrade chloroplast-derived proteins, including those encoding cysteine proteases, and vacuolar processing enzymes [52, 53]. *NtCP1* is only expressed in senescent tobacco leaves and was not induced in mature green leaves or by abiotic stress. *SAG12*, expressed in mature and senescent leaves, can also be used as a leaf senescence marker in tobacco [54]. Cysteine proteinases (*NtCP1* and *SAG12*) are important for the degradation of photosynthetic proteins such as Rubisco and Rubisco activase [55]. *NtCP1* and *SAG12* were induced indicating that senescence had started in transgenic plants.

We made a preliminary analysis of the effect of leaf senescence on the terpenoid synthesis pathway. Monoterpene production is inhibited under oxidative stress [56]. Monoterpenes, including β-Myrcene and linalool, have lower production in transgenic plants compared to wild-type plants (Fig. 1). In addition, carotenoid biosynthesis had 34 downregulated unigenes, geranylgeranyl reductase and solanesyl diphosphate synthase are downregulated in the MEP pathway (Additional file 3). The release of farnesene can reduce the supply of substrate for other terpenes in the MVA pathway such as caryophyllene and sterols. Sterols are essential for plant development and growth. This may be an additional reason for the senescence phenotype.

## Conclusion

The analysis of differentially expressed unigenes, physiological and biochemical parameters related to senescence highlighted that ROS play an important role in the senescence of transgenic plants. This study also indicates that Illumina sequencing technology can be applied as a rapid method for de novo transcriptome analysis of tobacco with unavailable genomic information.

## Methods

### Plant materials and treatments

Seeds (*Nicotiana tabacum* NC89) of WT, T3-1, T3-2 and T3-3 were germinated on Murashige–Skoog (MS) agar medium, in closed glass dishes for 14 days at 25/20 °C day/night cycle and 16:8 (L:D) h photoperiod. Seedlings were transplanted into vermiculite and grown at 25–30 °C/20–25 °C (day/night temperature regime) under a 16:8 h photoperiod 300–700 μmol m^−2^ s^−1^ photon flux density (PFD) and 50–60 % relative humidity in a greenhouse. Leaf samples were obtained from 8-week-old plants for subsequent experiments. At 8 weeks, the transgenic plants are still in vegetative growth. So the plants were considered to be at similar developmental stages. The measurements of physiological and biochemical parameters were conducted on the youngest fully expanded leaves. Five biological repeats were used in physiological and biochemical experiments.

For reagent treatment, seeds of WT, T3-1, T3-2 and T3-3 were sown into vermiculite and grown in an illuminated incubation chamber (GXZ-260C). Three week-old WT and transgenic plants were sprayed with 70 μM 3-(3,4-dichlorfenyl)-1,1-dimethylkarbonyldiamid (DCMU).

### Transformation and identification of transgenic tobacco plants

Based on the cDNA sequence of *MdAFS1* (GenBank accession number AY182241), a specific primer was designed (Additional file 6). Total RNA was isolated from apple peel of ‘*white winter pearmain*’ using Trizol (Tiangen, Beijing, China). DNA-free total RNA was reverse-transcribed using a RevertAid First-strand cDNA Synthesis Kit (MBI Fermentas, Beijing, China) according to the manufacturer’s protocol. The *MdAFS1* gene was isolated from apple (*white winter pearmain*). The open reading frame (ORF) of *MdAFS1* cDNA was inserted into the pBI122 expression vector under the control of the 35S Cauliflower mosaic virus promoter. The 35S-*MdAFS1* plasmid was introduced into *Agrobacterium tumefaciens* LBA4404, and was verified by PCR and sequencing. *N. tabacum* (NC89, saved by the current laboratory) was transformed with the resultant plasmid using the standard *Agrobacterium*- mediated method [57]. Three independent third generations lines (T3-1, T3-2, T3-3) were selected for further experimentation.

### Samples and analytical conditions for GC–MS analysis

Each 1.0 g fresh tobacco flower sample was extracted with 6 ml of extraction buffer supplemented with 50 mM KCl and 10 mM MgCl_2_ in a sealed container [58, 59]. The volatile compounds were collected with solid-phase microextraction (SPME) for 40 min at 40 °C.

Volatile compound analysis was performed with GCMS-QP2010 with a FID detector (Shimadzu, Tokyo, Japan). A Rtx-5MS fused-silica column (30 m × 0.32 mm × 0.25 μm) was used. The oven temperature was initially held for 2 min at 35 °C, ramped at 6 °C/min up to 120, 10 °C/min up to 180 and 20 °C/min up to 230 °C. Split injection (5:1) was used. The carrier gas was helium with a flow rate of 2.2 ml/min. Injector and FID detector temperature were held at 250 and 280 °C, respectively.

*Qualitative method* The results were analyzed using standard NIST08 gallery and spectra.

*Quantitative method* The results were analyzed using the normalization of peak area method.

### Measurement of pigment content

Pigment was extracted from fresh leaf samples (≈0.1 g) using 80 % acetone at room temperature in darkness until the leaf tissue was completely bleached. The extract was centrifuged at 5000*g* for 5 min, and the supernatant was collected and used in spectrophotometric analysis with a spectrophotometer (UV-1780, Shimadzu, Tokyo, Japan) at an absorbance wavelength of 470, 646 and 663 nm. The chlorophyll concentration was calculated using the following formula: chlorophyll a (C_a_) = 12.21*A_663_ – 2.81*A_646_; and chlorophyll b (C_b_) = 20.13*A_646_ – 5.03*A_663_. The total chlorophyll (a + b) content (mg g^−1^ FW) was then calculated. The carotenoid concentration (C_x*c_) was calculated using the following formula: carotenoids = (1000*A_470_ – 3.27*C_a_ – 104*C_b_)/229.

### Photosynthetic gas exchange measurements

CO_2_ gas exchange was measured between 9:00 and 11:00 h on the same day using a portable photosynthesis system (CIRAS-2, PP Systems, Herts, UK). Experiments were performed under the following conditions: greenhouse temperature (25 ± 1 °C); CO_2_ concentration, 390 μl l^−1^; PFD, 1200 μmol m^−2^ s^−1^, and relative humidity, 70–80 %. Irradiance was controlled by the automatic control function of the CIRAS-2 photosynthetic system.

### Physiological assays for leaf senescence

Each 0.5 g leaf sample was homogenized in 5 ml of 50 mM sodium phosphate buffer (pH 7.8) supplemented with 1 mM EDTA and 2 % (w/v) polyvinylpyrrolidone (PVP). The homogenate was centrifuged at 12,000*g* for 20 min at 4 °C; the supernatant was immediately used for the determination of O_2_^·−^ radical production rate, soluble protein content, and antioxidant enzyme activities. All assays were performed at 4 °C. Superoxide dismutase (SOD), catalase (CAT), and ascorbate peroxidase (APX) activities were determined as previously described [60]. Total soluble protein content was determined according to Bradford using bovine serum albumin (BSA) as the standard [61]. The assay for O_2_^·−^ content was conducted as described by Yang [62].

H_2_O_2_ content was measured according to Gay and Gebicki [63]. Tobacco leaves (0.5 g) were ground with liquid nitrogen, then transferred to a centrifuge tube, to which 2 ml of cold acetone was added. After centrifugation at 10,000*g* for 10 min, 1 ml of supernatant and 3 ml of 20 % titanium tetrachloride (TiCl_4_) were centrifuged at 4000*g* for 15 min. Twenty percentage (v/v) H_2_SO_4_ was added to the precipitate, dissolved, and the absorbance was determined at 410 nm.

Lipid peroxidation was determined by estimating malondialdehyde (MDA) content [64]. A 10 % solution of trichloroacetic acid (TCA) containing 0.6 % 2-thiobarbituric acid (TBA) and heated at 95 °C for 15 min, and the absorbances were determined at 450, 532 and 600 nm. All spectrophotometric analyses were performed using a spectrophotometer (UV-1780, Shimadzu, Tokyo, Japan).

Water content and seed biomass were determined using a drying oven (Yiheng, Shanghai, China). Fresh leaf samples were dried at 105 °C for 30 min, and 60 °C for 48 h. After collection, seeds were heated at 35 °C to constant weight, and the tobacco seeds were used in biomass statistics.

### Histochemical staining of H_2_O_2_ and O_2_^·−^

H_2_O_2_ and O_2_^·−^ accumulations were detected using 3,3′-diaminobenzidine (DAB) and nitroblue tetrazolium (NBT) staining methods [65, 66], respectively. H_2_O_2_ reacts with DAB to form a reddish-brown stain. Tobacco leaf discs (1.4 cm diameter) were incubated in 1 mg/ml DAB solution in the dark at room temperature for 16 h. Leaf discs were boiled in ethanol (95 %) for 10 min and then cooled to room temperature. The leaf discs were then extracted with fresh ethanol and photographed. O_2_^·−^ reacts with NBT to form a blue stain. A 0.5 mg/ml NBT solution was used in this experiment. The procedure used was similar to H_2_O_2_ staining.

### Transcriptome analysis

The tobacco plants were grown under greenhouse conditions as described previously. Samples were collected from 8-week-old plants. To produce a transcriptome dataset with a wide coverage, RNA was extracted from pooled samples of leaves from four plants of WT and T3-2, respectively. The high-quality reads were assembled with the software package Velvet_1.2.10. The trimmed Solexa transcriptome reads were mapped onto the unique consensus sequences using Bowtie. Unigenes were compared to records in public databases, including the National Center for Biotechnology Information (NCBI, 2013), SWISS-PROT, kyoto encyclopedia of genes and genomes database (KEGG), and gene ontology (GO). Transcriptome sequencing was performed by Capital Bio Corporation (Beijing, China), using Hiseq 2000.

### qRT-PCR analysis

Sampling from 8-week-old plants under the same greenhouse conditions as previously described, qRT-PCR analysis was conducted according to the MIQE guidelines [67, 68]. Total RNA was isolated using a total RNA isolation system (Tiangen, Beijing, China). First-strand cDNAs were synthesized using a First-strand cDNA Synthesis Kit (Tiangen, Beijing, China). qRT-PCR was performed on a Bio-Rad CFX96TM Real-time PCR System using SYBR Real Master Mix (Transgen, Beijing, China) using the following PCR thermal cycle conditions: pre-denaturation at 95 °C for 30 s; followed by 40 cycles of 95 °C for 5 s, 60 °C for 15 s, and 72 °C for 20 s. 18S RNA (GenBank Accession Number: AJ236016) was used as housekeeping gene. Three biological replicates were performed for each line, and the standard curve method was used for statistical analysis.

### Statistical analysis

Statistical analyses were performed using SigmaPlot 12.0 software, Excel, and data processing system (DPS) procedures (Zhejiang University, Zhejiang, China). Differences among means were compared using Tukey’s multiple range test at a 0.05 probability level.

## Additional files

10.1186/s40659-016-0088-1 Validation of transcript levels of six candidate unigenes of the transcriptome by qRT-PCR. Upregulated unigenes of the transcriptome database: Locus_72229: *Nicotiana* *tabacum* glycine-rich protein precursor, Locus_4438: Ent-copalyl diphosphate synthase, Locus_712: Polyphenol oxidase; downregulated unigenes of the transcriptome database: Locus_10435: Inositol-3-phosphate synthase, Locus_5382: Ethylene-responsive transcription factor 5, Locus_93559: Calvin cycle protein. The gene names and primers used for qRT-PCR analysis are shown in Additional file. 1. The standard error of the mean of three biological replicates (nested within three technical replicates) is represented by error bars.
10.1186/s40659-016-0088-1 Differentially expressed and identified unigenes in photosynthesis, nitrogen metabolism, nutrient reservoir activity, antioxidant activity, ubiquitin-mediated proteolysis, cell growth, and death.
10.1186/s40659-016-0088-1 Differentially expressed and identified unigenes in secondary metabolism signaling pathways.
10.1186/s40659-016-0088-1 Antioxidant metabolite levels of the WT and transgenic plants.
10.1186/s40659-016-0088-1 The relative values of GS, Pn, Ci, in tobacco leaves.
10.1186/s40659-016-0088-1 List of primers used for qRT-PCR analysis.

## Abbreviations

APX: ascorbate peroxidase
CAB: chlorophyll *a/b* binding protein
CAT: catalase
CHL: chlorophyllase
DAB: 3,3ʹ-diaminobenzidine
GDH: glutamate dehydrogenase
GO: gene ontology
GS2: chloroplastic glutamine synthetase
GC/MS: gas chromatography–mass spectrometry
H_2_O_2_: hydrogen peroxide
KEGG: kyoto encyclopedia of genes and genomes
LSD: leaf senescence database
MDA: malondialdehyde
MS: Murashige–Skoog
NBT: nitroblue tetrazolium
Nia: nitrate reductase
NtCP1: cysteine protease
Ntdin: a tobacco senescence-associated gene
O_2_^−^: superoxide anion
POD: peroxidase
qRT-PCR: quantitative real time-polymerase chain reaction
RBCS2B: RuBisCO small subunit
ROS: reactive oxygen species
SAG12: cysteine protease
SOD: superoxide dismutase
WT: wild-type
